# Unlocking the Potential: Phytoestrogens and Cardiovascular Health

**DOI:** 10.2174/011573403X333952241203050033

**Published:** 2025-01-02

**Authors:** Arvind Gulati, Himanshi Banker, Alina Amin Muhammad, Fnu Anamika, Rohit Jain

**Affiliations:** 1 Department of Internal Medicine, Maulana Azad Medical College, New Delhi, India;; 2 Department of Internal Medicine, Liaquat National Hospital and Medical College, Karachi, Pakistan;; 3 Department of Internal Medicine, University of Delhi, New Delhi, India;; 4 Department of Internal Medicine, Penn State Milton S. Hershey Medical Center, Hershey, PA 17033, USA

**Keywords:** Phytoestrogens, cardiovascular disease, congestive heart failure, thrombogenic reactions, myocardial infarction (MI), arrhythmias

## Abstract

Phytoestrogens are plant-derived compounds resembling human estrogen and have recently gained attention due to their potential role in improving cardiovascular health. These compounds exert their effects through various mechanisms, including interactions with estrogen receptors, growth factor receptors, inflammatory mediators, thrombogenic reactions, and apoptotic pathways. This results in cardioprotective effects like modulating endothelial function, decreasing vessel tone, reducing inflammation, altering lipid profiles, and influencing arrhythmogenesis. Recent studies indicate the intricate and multidimensional association between phytoestrogens and cardiovascular disease. Despite the overwhelming evidence that phytoestrogen intake lowers the risk of myocardial infarction (MI), prevents atherosclerosis, improves cardiac function, prevents hypertension, and reduces the risk of arrhythmias, there have been studies that show contradictory outcomes. For this reason, the therapeutic use of phytoestrogens for the treatment of cardiovascular diseases, which appears to be extremely promising, should be handled cautiously, considering the individual variances, dosage, and the specific components of phytoestrogens. This review consolidates findings on the effects of phytoestrogens on the heart and blood vessels, explores the mechanisms behind these interactions, and seeks to determine the best methods for using phytoestrogens as a supplement in managing and preventing cardiovascular disease. By understanding these aspects, we can better evaluate the potential of phytoestrogens in cardiovascular health and develop guidelines for their safe and effective use.

## 1 INTRODUCTION

Phytoestrogens are a group of phenolic compounds resembling estrogens, specifically 17-β-estradiol (E2), found in plants and plant-based products, especially soy. The most common phytoestrogens are isoflavones and their significant derivatives like aglycones, daidzein, Genistein, and glycitein, with Genistein being the predominant derivative found in soybeans [1, 2]. Being considered natural alternatives to estrogen, phytoestrogens have gained popularity for treating menopausal symptoms, such as hot flushes and osteoporosis. In addition to treating menopausal symptoms, phytoestrogens have been proven beneficial for cardiovascular health in females, particularly postmenopausal females.

Cardiovascular diseases are considered the foremost cause of morbidity and mortality, with WHO estimating that around 23.6 million deaths worldwide will be related to CVDs [3]. Among the various nonmodifiable risk factors for CVD, gender plays a significant role in determining the mortality risk in the population. Before the age of 50, women have a less likely risk of CVD compared to men, but as they age, the prevalence rises dramatically, reaching nearly the same level as in men by the time they reach their seventh decade of life [4]. It is often hypothesized that estrogen usually offers a protective role over the cardiovascular system. As age advances with loss of ovary reserve, estrogen levels decrease and often pose a significant challenge to cardiovascular health due to the alteration in lipid profile with an increase in LDL and decrease in HDL levels. For this reason, besides traditional risk factors (smoking, obesity, metabolic syndrome, hypertension, physical inactivity, high lipids, and diabetes mellitus) of CVD, in females, the nontraditional factors generally associated with pregnancy, such as preeclampsia, gestational hypertension, gestational diabetes mellitus, menopause-related hormonal changes, pose a significant threat to female cardiovascular health [4].

Since estrogen often offers a protective role over cardiovascular health, it is postulated that increased use of estrogen, particularly the phytoestrogens, may provide protective cover to cardiovascular health as various *in vitro* studies have demonstrated that phytoestrogens reduce arterial stiffness and generate NO, a potent vasodilator [5, 6]. In addition, various *in vivo* studies have documented that phytoestrogens positively affect thrombosis, vascular reactivity, lipid profiles, and cellular proliferation [7]. On the contrary, various clinical studies have failed to demonstrate any positive effect of phytoestrogen on cardiovascular disease in clinical settings [8]. Studies have further suggested that isoflavones did not affect serum lipid levels, and natural soy products seem to have a higher hypolipidemic effect on serum cholesterol than soy supplements [9].

This narrative review aims to summarize the most recent research on the connection between dietary phytoestrogens and cardiovascular disease. It aims to discuss the relationship between phytoestrogens and their role in the prevention and management of cardiovascular disease (CVD) by explaining the pathophysiology of their possible cardioprotective effects, clarifying their influence on cardiovascular risk factors, and analyzing clinical evidence from the available literature.

## 2 PATHOPHYSIOLOGY

Phytoestrogen affects multiple organ systems *via* a number of different mechanisms; however, most of the effects are through its effect on estrogen receptors. Other mechanisms of action are mediated by interfering with the growth factor receptor, inhibition of the G protein-coupled receptor in ER-deficient cells, and activation of apoptosis [10]. The impact of phytoestrogens varies among different individuals. Factors, such as the type and quantity of exposure, bioavailability, ethnic background, hormonal status (age, gender, and physiological condition), microbiota composition, and the health status of the individual consumer can lead to differences in its beneficial effect [11].

The gut microbiota plays a crucial role in metabolizing phytoestrogens, impacting their bioavailability, which can vary among individuals due to differences in microbiota composition. Phytoestrogens are initially present as inactive glycosidic conjugates in plant sources, which are then metabolized by UDP-glucuronosyltransferase enzymes produced by intestinal bacteria [12]. This process results in the conversion of inactive conjugates into active aglycones, with genistein and daidzein being the most active forms of isoflavones. These active metabolites enter circulation and exhibit functional similarities to estradiol (E2), thereby inducing (anti)estrogenic effects in the body. Subsequently, the liver conjugates these metabolites, producing β-glucuronides, which are eliminated through bile [13]. Notably, certain gut bacteria, such as lactic acid bacteria and bifidobacteria, have a tendency to convert genistein and daidzein into genistein, contributing to variations in this metabolic process [5].

Phytoestrogens exhibit cardioprotective effects through various mechanisms, with a prominent one being their influence on the vascular endothelium. Estrogenic effects, mediated by ERα (Estrogen receptor α) and ER β (Estrogen receptorβ), involve ERα promoting cell proliferation while ERβ acts as a regulatory counterbalance to ERα [14]. Phytoestrogens, being less potent than E2, demonstrate a higher affinity for ERβ over ERα. Similar to E2, phytoestrogens regulate endothelial cell (EC) proliferation, maintain EC integrity, and reduce vascular permeability. Moreover, they impact vascular tone significantly. ERs facilitate endothelium-dependent vascular relaxation, with E2 promoting the production of NO (nitric oxide), PGI2 (prostaglandin I2), and EDHF (endothelium-derived hyperpolarizing factor) while decreasing ET (endothelin) release. Phytoestrogens promote endothelium-mediated vascular relaxation similar to E2. Like E2, they enhance eNOS expression in human ECs by augmenting eNOS promoter activity, along with increasing EC Ca^2+^, MAPK, and PI3K activity, consequently elevating eNOS activity and NO production [12]. Genistein-induced coronary vasodilation also involves ERα/ERβ and stimulation of β2-adrenoreceptors [15]. The production of NO results in the activation of guanylyl cyclase, leading to increased production of cGMP in vascular endothelial cells, ultimately causing vasodilation. This E2-induced increase in cGMP is notably heightened in ischemic tissue, contributing to the cardioprotective role of phytoestrogens by delaying ischemic damage. Additionally, phytoestrogens may also enhance adenylate cyclase activity and impact cAMP-dependent pathways in endothelial cells (ECs) and vascular smooth muscle cells (VSM), similar to prostaglandins [13].

The anti-inflammatory response of phytoestrogens operates through the mediation of the nuclear factor-kappa B (NF-κB) signaling pathway. External stimuli, such as tumor necrosis factor-alpha (TNF-α), trigger the activation of IκB kinase (IKK), leading to the release of NF-κB in inflamed tissues. NF-κB subsequently translocates to the nucleus, regulating the transcription of pro-inflammatory genes. Phytoestrogens function by inhibiting this pathway, thereby suppressing inflammation and exerting an anti-inflammatory effect on the myocardium [16]. Furthermore, research suggests that phytoestrogens may inhibit fibroblasts regulated by transforming growth factor-beta (TGF-β), potentially reducing fibrosis and mitigating myocardial damage in cases of ischemia and oxidative stress [7]. This anti-inflammatory effect not only protects myocardial cells from damage but may also contribute to the reduction of atherogenesis.

Additionally, phytoestrogens may exert anti-atherogenic effects by altering lipid profiles. The hypocholesterolemic effects of phytoestrogens are multifactorial, including an increase in bile acid excretion, leading to enhanced removal of low-density lipoprotein (LDL), and potentially altering hepatic metabolism to augment LDL and very low-density lipoprotein (VLDL) removal by hepatocytes. Moreover, phytoestrogens may protect against atherosclerosis by decreasing intercellular signaling and oxidant-sensitive transcription of adhesion molecules responsible for the lipid-induced cascade of monocyte-predominant inflammation in atherosclerotic blood vessels [17]. Therefore, a diet rich in Iso flavonoids can be considered a lipid-lowering diet and may be recommended for patients with coronary artery disease (CAD).

The anti-arrhythmogenic effects of phytoestrogens, although less explored, offer significant cardioprotective benefits. Research indicates that genistein, a phytoestrogen, has the potential to enhance the sensitivity of the cAMP-regulated Cl^-^ current to β-adrenergic stimulation. β-Adrenergic regulation of ion channels is a widespread mechanism for controlling both cardiac electrical and mechanical activity, facilitated by the inhibition of tyrosine kinase activity in myocytes [18]. Phytoestrogen-induced tyrosine kinase inhibition has also been found to effectively reduce thrombin-evoked protein tyrosine phosphorylation in human platelets. The inhibitory effects of genistein on thrombogenesis and platelet aggregation are comparable to those of acetylsalicylic acid. This multifaceted effect of tyrosine kinase inhibition by phytoestrogens presents opportunities for various cardiovascular therapeutics with minimal adverse effects [7]. A summary of the potential mechanisms by which phytoestrogens exert a cardio-protective effect is illustrated in Fig. (**1**).

## 3 DISCUSSION

Phytoestrogens and cardiovascular disease have a complicated and multidimensional interaction. It is crucial to remember that results from certain studies have been conflicting or unclear, and the overall effects could vary depending on the particular phytoestrogen chemical, the dose, the length of exposure, and personal factors, including age, sex, and metabolic condition.

Phytoestrogens have been correlated with a decrease in the incidence of myocardial infarction and angina. In a recent study published by Barsky *et al*. in 2021, 143 women were followed for phytoestrogen levels and 10-year outcomes. It was found that women with low genistein had an increased frequency of major adverse cardiac events, including myocardial infarction and early hospitalization for angina after 6 years. The study results showed that the incidence of MACE rose after six years for every unit drop in log genistein (HR 6.17, 95% (CI 1.81, 20.8), *p* = 0.0036) [19]. Other studies conducted in the past have also shown an inverse relationship between phytoestrogen intake and CHD. In a cohort study of 64,915 Chinese women conducted by Zhang *et al*., after a mean follow-up period of 2.5 years, the relative risk of CHD in women with the highest quartile for soy consumption as compared to women in the lowest quartile was 0.25 with a 95% CI of 0.10 - 0.63. [20]. The WHO CARDIAC (Coordinated Cardiovascular Diseases and Alimentary Comparison) Study examined 24-hour urine soy isoflavones among 61 populations in 25 countries, providing ecological evidence of a strong inverse correlation between soy isoflavones and CHD mortality. Of the populations included in this study, it was observed that Japanese had the highest 24-hour excretion of isoflavones and the lowest age-adjusted mortality rate of CAD [21]. Similarly, Kokubo *et al*. concluded that a high isoflavone intake was linked to a lower incidence of MI, and the risk reduction was more marked for post-menopausal females. The Hazard Ratio for MI in women comparing the highest versus the lowest quintiles of isoflavone consumption was calculated to be 0.37 (0.14 to 0.98). However, their study did not show any significant association between intake of isoflavones and MI in men [22]. This gender-specific difference in the cardioprotective effects of phytoestrogens can be explained by findings from a study conducted by Pan *et al*. Their study demonstrated that phytoestrogens were associated with an increased risk of metabolic syndrome in males compared to females. Since metabolic syndrome is a key predictor of cardiovascular health, this elevated risk in men may counteract the potential benefits of phytoestrogens on cardiovascular outcomes, thus explaining the lack of significant protective effect in males [23]. In contrast, a study conducted by van der Schouw *et al*. on a sample of 16,165 European women had a contradictory finding and reported no connection between consumption of isoflavones and CVD [24]. The average diet in East and Southeast Asia is thought to contain 20–50 mg of phytoestrogen per day, while Western countries have substantially lower intakes because of their limited usage of soy products. Intakes in the US have been documented to range from 0.15 mg/d to more than 3 mg/d. According to data about European nations, average values for men and women range from 0.63 to 1.00 mg/d and 0.49 to 0.66 mg/d, respectively [25]. Therefore, the contrasting finding of the study conducted by van der Schouw *et al*. can possibly be explained by the low dietary intake of isoflavones in the European population.

An RCT conducted by Hodis *et al*. studying the effect of soy protein supplementation on carotid intima-media thickness, which is widely used as a surrogate marker of atherosclerosis, showed that the mean carotid intima-media thickness progression rate was 4.77 µm/yr. in postmenopausal females receiving 25 g soy protein supplementation as compared to 5.68 µm/yr. in the placebo group, suggesting a 16% reduction in Carotid intima-media thickness (cIMT). However, the results of this study were not statistically significant (*p*= 0.36). However, a subgroup analysis in the same study showed that in women within five years of menopause, soy protein supplementation slowed the progression of subclinical atherosclerosis, which was clinically significant [26]. These findings were supported by a study conducted in 2016 in post-menopausal females using a phytoestrogen-rich herbal preparation containing 500 mg of Iso flavonoids, which showed significant inhibition in the progression of cIMT as compared to the placebo group. This study also showed that in receivers of Iso flavonoid-rich herbal preparations, the growth of pre-existing atherosclerotic plaques was slowed by 1.5 times [21]. Similarly, a cross-sectional study on a sample of 272 Japanese men found that the odd’s ratio for the presence of Coronary Artery Calcium (CAC) greater than 10 Agatson units in equol producers compared to non-producers was 0.10 (95% CI of 0.01- 0.90; *p*<0.04). [27]. The results of these investigations imply that phytoestrogens may have the ability to prevent the formation of new atherosclerotic lesions as well as to slow the progression of pre-existing ones.

The results of studies investigating the effect of phytoestrogens on Blood pressure have been conflicting.   Taking isoflavone-rich jelly for five weeks was found to significantly lower blood pressure in the isoflavone group compared to the placebo group in the WHO-CARDIAC/MONALISA research, which involved sixty Scottish women who had reached menopause [28]. On the other hand, a 2019 meta-analysis by Hemati *et al*. revealed that genistein had no discernible impact on SBP and DBP [29]. Similarly, an RCT conducted by Hodgson *et al*. discovered that Isoflavonoid supplementation in the form of 55 mg tablet, including 30 mg of genistein, 16 mg of biochanin A, 1 mg of daidzein, and 8 mg of formononetin for 8 weeks, did not lower BP in subjects with ambulatory BP recordings in normotensive range. Based on these findings, it was suggested that other dietary components in phytoestrogen-rich foods may be necessary for daidzein and genistein to exhibit BP-lowering effects [30].

Although not well studied for their effects on heart rhythm and function in humans, phytoestrogens have been shown to have antiarrhythmic effects as well as positive effects on heart failure in animal studies. Resveratrol has been shown to decrease left atrial fibrosis and atrial fibrillation susceptibility in rabbits with failing hearts [31]. Qin *et al*. induced pressure overload on the heart of mice using transverse aortic constriction. After 8 weeks, it was observed that mice who were given genistein had a significantly lower decline in ejection fraction. Additionally, the hearts of these mice grew noticeably less in size when exposed to pressure overload, demonstrating the capacity of genistein to prevent cardiac hypertrophy [32].

Hoie *et al*. demonstrated that supplementing with isolated soy protein can significantly lower plasma concentrations of total and low-density lipoprotein cholesterol. This study also demonstrated that the preservation of the original protein structure in isolated soy protein leads to more noticeable decreases in cholesterol levels, highlighting the importance of processing methods in retaining cardiovascular benefits [33]. A meta-analysis of 16 studies conducted in 2022 concluded that genistein supplementation significantly lowered LDL-C and total cholesterol levels in postmenopausal women, suggesting a potential therapeutic role in cardiovascular disease management. However, the meta-analysis showed no improvements in HDL and TG [34]. Furthermore, a 3-month study in normotensive men and postmenopausal women showed that supplementing with dietary soy led to an increase in Lp(a), a pro-atherogenic lipid, by 15% [35]. Similarly, a study conducted in Brazil reported no significant changes in total cholesterol, triacylglycerol, VLDL, LDL, and HDL concentrations following isoflavone supplementation compared to a casein placebo [36]. These conflicting results emphasize the need for further research to elucidate the effects of phytoestrogens on lipid levels.

These results are summarized in Table **1**.

## CONCLUSION

The complex relationship between phytoestrogens and cardiovascular disease offers a diverse range of management opportunities. Phytoestrogens, mimicking the actions of endogenous estrogens, exert their effects primarily through interactions with estrogen receptors, particularly ERβ, impacting various cellular pathways. They exhibit diverse cardioprotective mechanisms, including modulation of vascular endothelium proliferation and vessel tone, anti-inflammatory action, and alteration of lipid profiles.

Although some studies are still inconclusive, clinical trials have shown that phytoestrogens can prevent MI and may also lower blood pressure, improve heart function, prevent arrhythmias, and halt the development of atherosclerosis. While promising, the therapeutic application of phytoestrogens in the treatment of cardiovascular disease should be approached with caution, considering factors, such as dosage, individual differences, and the particular components of phytoestrogens. To determine the most effective strategies for using phytoestrogens as an adjuvant medicine in the prevention and treatment of cardiovascular disease, more extensive clinical studies and investigations are required.

## Figures and Tables

**Fig. (1):**
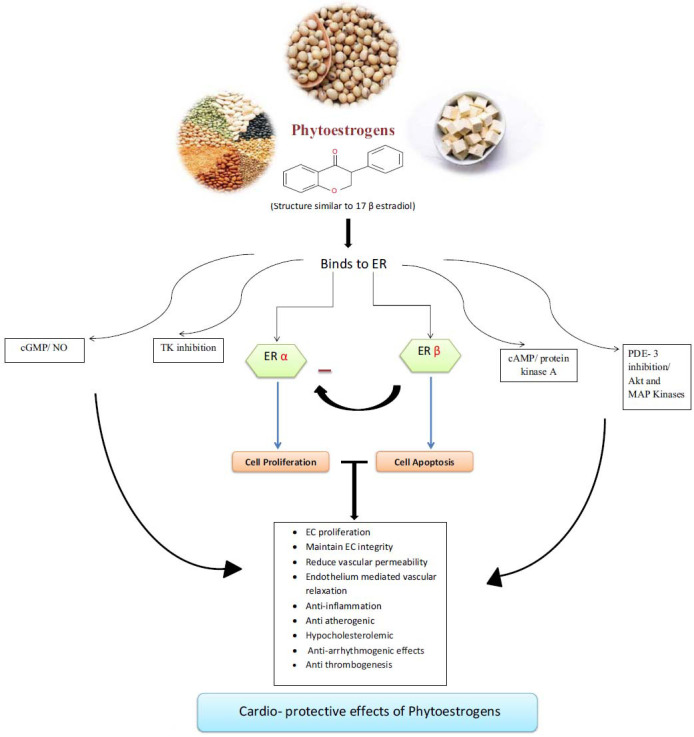
Illustrates all the potential mechanisms by which phytoestrogens may exert their cardio-protective effects. **Abbreviations:** ER (estrogen receptor); cGMP (cyclic guanosine monophosphate); TK (Tyrosine kinase); cAMP (cyclic adenosine monophosphate); PDE (phosphodiesterase 3); Akt (protein kinase B); MAP (mitogen activated protein).

**Table 1:** Effects of phytoestrogens on lipid levels.

**S. No.**	**Name**	**Study Design**	**Parameter Studied**	**Conclusion**
1.	Barsky [22]	Prospective cohort study	Major adverse Cardiac Events (MACE) including MI, stroke, hospitalization for heart failure and angina	At six years, reduced genistein was linked to significantly higher risk of MACE.
2.	Kokubo [23]	Prospective cohort study	Myocardial Infarction	Postmenopausal women showed a significant inverse relationship between isoflavone intake and risk of CI and MI. In men, there was no discernible link between isoflavone dietary consumption and MI.
3.	Dutch Prospect-EPIC cohort (European Prospective study Into Cancer and nutrition) [24]	Prospective cohort study	Coronary heart disease	No evidence that high intake of phytoestrogens leads to a significant decrease in risk of coronary heart disease in European women.
4.	Hodis [26]	Randomized control trial	Carotid intima-media thickness (CIMT)	Isoflavone supplementation did not lead to a statistically significant decrease in CIMT progression in postmenopausal females. However, subgroup analysis showed that the effect was significant in women who had their menopause within 5 years.
5.	Myasodeva [27]	Randomized control trial	CIMT	Isoflavonoid rich herbal preparation lead to a significant decrease in mean CIMT progression in postmenopausal females.
6.	WHO-CARDIAC/MONALISA study [28]	Randomized control trial	Blood pressure	A significant reduction in BP was seen in isoflavone taking group as compared to placebo group.
7.	Hemati [29]	Meta-analysis	Blood pressure	Genistein does not show any statistically significant effect on SBP and DBP.
8.	Chong [30]	Randomized control trial in rabbits	Left atrium fibrosis and atrial fibrillation susceptibility	Resveratrol decreases LA fibrosis and atrial fibrillation susceptibility in rabbits with failing hearts
9.	Wei Qin [31]	Randomized control trial in mice	Ejection Fraction	Genistein prevents a decline in ejection fraction and prevents cardiac hypertrophy in hearts exposed to pressure overload.
10.	Hoie [32]	Randomized control trial	Total cholesterol, LDL	Soy protein in its native protein state leads to a pronounced decrease in Total cholesterol and LDL.
11.	Amerizadeh [33]	Meta-analysis	Triglycerides, Total cholesterol, LDL, HDL, SBP and DBP	Consuming genistein dramatically lowers LDL, SBP, and total cholesterol. Triglycerides, HDL, and DBP do not significantly improve.
12.	Teede [34]	Randomized control trial	BP, LDL/HDL, TG, Lp(a), Total cholesterol, LDL and HDL	Soy protein isolate results in significant fall in BP, LDL/HDL ratio and Triglycerides. It leads to increase in Lp(a). No significant effect was seen on total cholesterol, LDL and HDL

## LIST OF ABBREVIATIONS

CVD: Cardiovascular Disease
EC: Regulate Endothelial Cell
ECs: Endothelial Cells
MI: Myocardial Infarction
VSM: Vascular Smooth Muscle Cells
